# The CSN3 subunit of the COP9 signalosome interacts with the HD region of Sos1 regulating stability of this GEF protein

**DOI:** 10.1038/s41389-018-0111-1

**Published:** 2019-01-04

**Authors:** Natasha Zarich, Begoña Anta, Alberto Fernández-Medarde, Alicia Ballester, María Pilar de Lucas, Ana Belén Cámara, Berta Anta, José Luís Oliva, José M. Rojas-Cabañeros, Eugenio Santos

**Affiliations:** 1 Unidad Funcional de Investigación de Enfermedades Crónicas (UFIEC) and CIBERONC, Instituto de Salud Carlos III, 28220 Majadahonda, Madrid Spain; 2 Centro de Investigación del Cáncer, IBMCC (CSIC-USAL) and CIBERONC, Universidad de Salamanca, 37007 Salamanca, Spain

## Abstract

Sos1 is an universal, widely expressed Ras guanine nucleotide-exchange factor (RasGEF) in eukaryotic cells. Its N-terminal HD motif is known to be involved in allosteric regulation of Sos1 GEF activity through intramolecular interaction with the neighboring PH domain. Here, we searched for other cellular proteins also able to interact productively with the Sos1 HD domain. Using a yeast two-hybrid system, we identified the interaction between the Sos1 HD region and CSN3, the third component of the COP9 signalosome, a conserved, multi-subunit protein complex that functions in the ubiquitin–proteasome pathway to control degradation of many cellular proteins. The interaction of CSN3 with the HD of Sos1 was confirmed in vitro by GST pull-down assays using truncated mutants and reproduced in vivo by co-immunoprecipitation with the endogenous, full-length cellular Sos1 protein*.* In vitro kinase assays showed that PKD, a COP9 signalosome-associated-kinase, is able to phosphorylate Sos1. The intracellular levels of Sos1 protein were clearly diminished following CSN3 or PKD knockdown. A sizable fraction of the endogenous Sos1 protein was found ubiquitinated in different mammalian cell types. A significant reduction of RasGTP formation upon growth factor stimulation was also observed in CSN3-silenced as compared with control cells. Our data suggest that the interaction of Sos1 with the COP9 signalosome and PKD plays a significant role in maintenance of cellular Sos1 protein stability and homeostasis under physiological conditions and raises the possibility of considering the CSN/PKD complex as a potential target for design of novel therapeutic drugs.

## Introduction

Ras proteins are small GTPases acting as molecular switches to connect various extracellular signals to intracellular pathways that modulate cellular proliferation, differentiation, senescence or death. They are continuously cycling between inactive (Ras-GDP) and active (Ras-GTP) conformations through a process modulated by negative (GTPase activating proteins, GAPs) and positive (guanine nucleotide-exchange factors, GEFs) regulators. The Sos1 and Sos2 proteins constitute the most universal and widely expressed family of RasGEFs in mammalian cells^1–6^.

The highly homologous, ubiquitously expressed Sos1 and Sos2 proteins^7,8^ exhibit a multi-modular structure featuring conserved distribution of specific, functional domains along their amino-, central-, or carboxy-terminal regions^1,5,6^. The carboxyl-terminal end of Sos proteins is a proline-rich (PR) region adopting left-handed type II helix conformation^9–11^, which binds the SH3 domains of Grb2^8,12,13^. Two distinct hSos1 isoforms differing in their Grb2-binding ability and biological potency have been identified in this region^14,15^. The amino-terminal region of Sos proteins includes the histone-like domain (HD), Dbl-homology domain (DH), pleckstrin domain (PH), and helical linker (HL)^6^. The Ras-Exchange motif (REM) and the CDC25-homology (CDC25-H) domains are located centrally, between the HL domain and the PR motif, and constitute the catalytic-center of the nucleotide-exchange activity on Ras proteins^16^. Interaction of the REM domain with the switch-2 region of Ras mediates binding to Ras-GDP whereas, simultaneously, connection of the two β-sheets of the CDC25H domain with the switch-1 region of Ras promotes dissociation of GDP^17^.

Various structural studies indicate that the amino-terminal region plays a role in allosterical regulation of the overall RasGEF activity of Sos^6,18,19^. This N-terminal region concentrates most of the activating (*gain-of-function*) Sos1 germline mutations detected in Noonan syndrome cells^20,21^, where hyperactivation of Ras is the result of reduced Ras-GEF self-inhibition^6^. The HD region shows high homology to histone H2A and contains two tandem histone folds^19^. Structural studies have shown that the HD stabilizes the self-inhibited conformation of Sos proteins through its basal conformational interaction with the DH-PH region^22^. In this conformation, the DH-PH domain blocks the Ras allosteric binding site^17,22^ and, only after the stimulation by growth factors, the complete activation of Sos may proceed. This activation requires growth-factor-induced generation of phosphatidic acid via phospholipase D2^23^ followed by recruitment of Sos to the plasma membrane through the PH domain^6^ and HD binding to phosphatidic acid, thus promoting Sos RasGEF activity in the plasma membrane^24^.

Based on the HD tertiary structure and its function as a modulator of Sos-GEF activity, we hypothesized that this domain could interact in vivo with some cellular proteins in order to regulate Sos RasGEF overall functionality. Here, we demonstrate in vitro and in vivo interaction between the HD of Sos1 and CSN3, a subunit of the highly conserved COP9 signalosome complex^25^ and also show experimental evidences supporting the functional relevance of this HD-mediated interaction, indicating that the COP9 signalosome and its associated PKD contribute to regulating the half-life and homeostasis of cellular Sos1 protein and therefore the activity of the Ras signaling pathway.

## Results

### CSN3 interacts with the HD of Sos1 in a yeast two-hybrid system

**Fig. 1:**
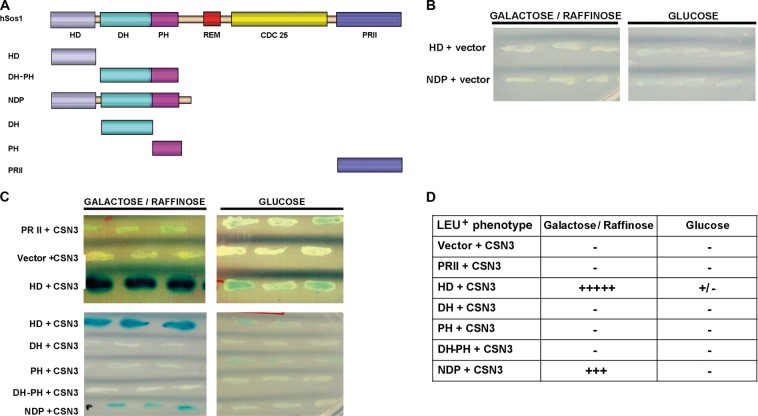
Interaction between CSN3 and hSos1 N-terminal fragments in a yeast two-hybrid system. **a** Scheme of the primary structure of the truncated fragments of hSos1. **b** The constructs in pEG202 coding respectively for the LexA fused to the HD or NDP and vector pJG4-5 alone (without CSN3) do not present β-galactosidase activity in X-Gal plates assays. Three independent colonies from each co-transformation were analyzed. **c** B42-activation domain fusion plasmid coding for B42-CSN3 (construct in pJG4-5) was co-transformed into yeast strain EGY48 (ura3, his3, trp1, LexAop-leu2) with the plasmid pSH18-34 (LacZ reporter with LexA-binding sites) and with LexA DNA binding domain fusion plasmids (constructs in pEG202) coding respectively for the LexA fused to the same HD, DH, PH, DH-PH, NDP fragments and for the PRII region of hSos1 (negative control). As others negative controls, plasmid pEG202 was co-transformed with pJG4-5-CSN3 (Vector + CSN3). Three independent colonies from each co-transformation were analyzed for β-galactosidase activity in color X-Gal plates assays. The specificity of the interactions was tested by dependence to galactose-raffinose. **d** LEU^+^ phenotype by patching these colonies in medium lacking leucine

To identify candidate cellular proteins able to interact with the amino-terminal region of hSos1, we performed yeast two-hybrid screens using a host strain (EGY48, harboring both LacZ and Leu reporter genes under control of LexA) that was co-transformed with a HeLa cDNA plasmid library (clones fused to B42-activation domain) and different plasmid baits coding for various truncated fragments of the N-terminal region of hSos1 (fused to LexA-DNA-binding domain) (Fig. 1a). As internal control, we confirmed previously that none of the hSos1 amino-terminal fragments tested could by itself and alone activate transcription of the reporter genes (Fig. 1b), and we also assessed that the LexA-fusion peptides correctly reached the nucleus of our yeast host strain EGY48 (data not shown).

Initial screening using a LexA-NDP plasmid bait coding for the first 548 amino-terminal residues of hSos1 yielded high numbers of positive, interacting clones. After successive rounds of screening, two clones showing positive interactions were selected for further study (Fig. 1c). After DNA sequencing, we found that in both cases the target cDNAs coded for the **C**OP9 **s**ig**n**alosome complex subunit **3** protein (CSN3) (NP_001303284.1).

In order to determine whether any specific subdomain within the NDP region was specifically responsible for the interaction with CSN3, we performed additional two-hybrid screenings testing the interaction between our positive cDNA clones coding different LexA-fused peptide subdomains (HD, NDP, DH, PH, DH-PH) of the NDP region (Fig. 1c). As internal control, we also tested in these assays a similar LexA-fusion construct coding for the C-terminal PRII region of hSos1, which is responsible for interaction with the Grb2 adaptor protein. Positive interactions occurring between CSN3 and the different peptide subdomains tested in these assays were revealed by the β-galactosidase activity (blue color) and the growth of the corresponding yeast clones in culture medium lacking leucine (Fig. 1c, d). Control clones co-transformed with both PRII and CSN3, or clones harboring CSN3 alone or the hSos1 N-terminal peptide subdomains alone, did not elicit any β-galactosidase activity (Fig. 1b, c) and did not grow on Leu^−^ plates (Fig. 1c, d). In contrast, transformants containing both CSN3 and HD showed high levels of β-galactosidase activity (Fig. 1c) and grew significantly on Leu^−^ medium (Fig. 1d). Judging by level of β-galactosidase activity and growth on Leu^−^ plates, the degree of interaction between CSN3 and the HD peptide was significantly high in the co-transformed yeast clones expressing both peptides, whereas the target–bait interaction appeared to be a bit weaker in those clones harboring CSN3 and the NDP peptide (Fig. 1c, d). In contrast, co-transformants harboring CSN3 and any of the DH, PH, DH-PH, or PRII peptides did not elicit any detectable β-galactosidase activity (Fig. 1c) and did not grow well on Leu^−^ plates (Fig. 1d). The specificity of our conclusions was validated by our use of an on/off model in these assays (β-galactosidase activity and LEU^+^ phenotypes detected only in yeast colonies grown on galactose-raffinose medium [on-state, induced expression of B42-CSN3]; and undetectable when growing on glucose medium [off-state, no expression of B42-CSN3]) (Fig. 1d).

These observations in a two-hybrid system strongly suggest that cellular CSN3 is able to bind to the amino-terminal region of hSos1, and that this molecular interaction specifically involves the HD region/domain of Sos1 protein.

### In vitro binding of HD and NDP peptides from the amino-terminal region of hSos1 to cellular CSN3

**Fig. 2:**
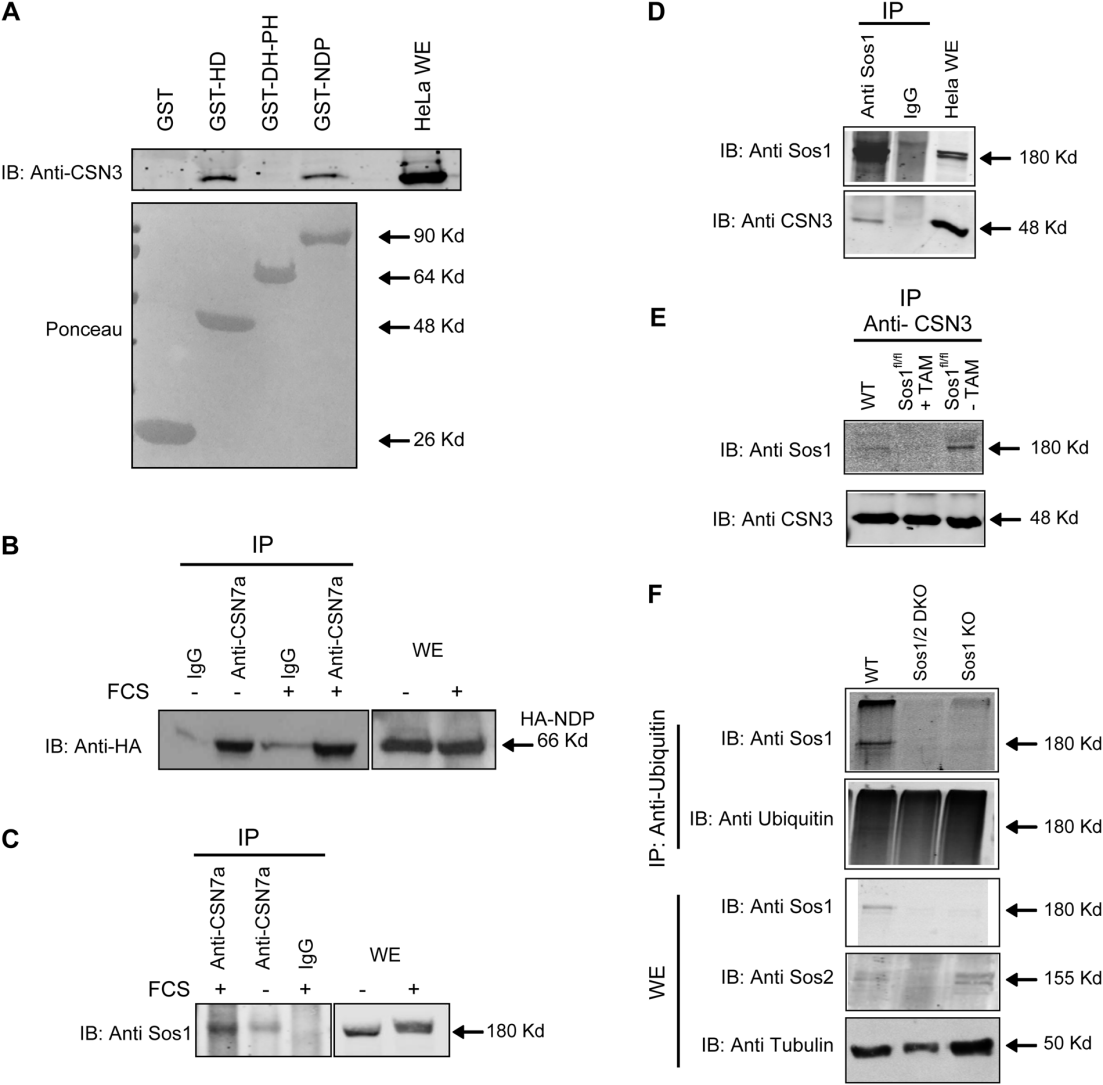
Sos1 protein interacts with the multi-protein complex COP9 signalosome. **a** Binding of cellular CSN3 to GST-DH-PH, GST-HD and GST-NDP fusion proteins in cell-free extracts. HeLa cell-free extracts were incubated with 20 μg of GST, GST-DH-PH, GST-HD, and GST-NDP proteins coupled to glutathione–sepharose beads. After washing, cellular proteins bound to the beads were resolved in SDS-PAGE alongside the corresponding cell extract and immunoblotted against anti-CSN3 antibodies. Lower part shows Ponceau staining showing the expression of GST, GST-HD, GST-DH-PH, and GST-NDP fusions bound to the membrane prior to western blot analyses. **b** In vivo interaction between the NDP region of hSos1 and the COP9 signalosome. Cell extracts from transiently transfected HeLa cells overexpressing NDP with HA epitope tag were starved for 16 h and stimulated with fetal calf serum for 10 min, then cell extracts were incubated with anti-CSN7a polyclonal rabbit antibody or with unspecific rabbit IgG. The anti-CSN7a immunoprecipitates or whole-cell extracts (WE) were then analyzed by immunoblotting using anti-HA monoclonal antibody as described in Materials and methods. **c** In vivo interaction of the endogenous full-protein Sos1 with the COP9 signalosome. Cell extracts from mouse embryonic cells Sos2-KO, serum deprived and stimulated with fetal calf serum for 10 min, were incubated with anti-CSN7a rabbit polyclonal antibody or with unspecific rabbit IgG. The anti-CSN7a immunoprecipitates or whole cell extracts were then analyzed by immunoblotting using anti-Sos1 mouse monoclonal antibody as described in Materials and methods. **d** Interaction between Sos1 and CSN3 in HeLa cells. Cell lysates or immunoprecipitates thereof generated using Sos1 antibodies or non-specific IgG were then immunoblotted (IB) with anti-Sos1 or anti-CSN3 antibodies, as indicated. **e** Interaction between Sos1 and CSN3 in mouse embryo fibroblasts (MEFs). Anti-CSN3 immunoprecipitates of lysates from wild type (WT) or Sos1^fl/fl^ primary MEFs that were kept untreated (-TAM) or treated (+ TAM) with tamoxifen in order to deplete Sos1 were submitted to western immunoblots (IB) using the indicated anti-Sos1 or anti-CSN3 antibodies. Pierce^®^ Crosslink Immunoprecipitation Kit (ThermoFisher) was used for panels **d** and **e**, in order to avoid signal interference from the immunoglobulin heavy chains. **f** Detection of Sos1 ubiquitination. Direct cell lysates (WE) or anti-ubiquitin immunoprecipitates thereof (IP: anti-Ubiquitin) obtained from wild type (WT), and tamoxifen-induced Sos1-KO or Sos1/2-DKO primary MEFs were further submitted to immunoblotting (IB) using anti-Sos1, anti-Sos2 or anti-Ubiquitin antibodies, as indicated

To directly validate our yeast two-hybrid findings and confirm the ability of CSN3 to bind to different hSos1 N-terminal fragments/domains, we carried out in vitro binding experiments using pull-down assays. Cytoplasmic extracts from HeLa cells were incubated with purified GST-HD, GST-DH-PH, and GST-NDP fusion proteins coupled to glutathione–sepharose beads that were subsequently tested by means of immunoblotting with antibodies against CSN3 (Fig. 2a). Whereas purified GST-DHPH did not bind any CSN3, the GST-HD and GST-NDP peptides bound roughly similar amount of cellular CSN3 in lysates of actively growing HeLa cells (Fig. 2a, upper panel). Ponceau staining (Fig. 2a lower panel) showed similar levels of the peptides in all cases, confirming that the HD and NDP regions of hSos1 are able to bind in vitro to CSN3 from cellular lysates, whereas the DH and PH domains of hSos1 do not possess such an interaction capability.

### In vivo binding of the cellular COP9 signalosome to individual peptides of the N-terminal region of hSos1 or the full-length, endogenous mSos1 protein

CSN3 is a component of the multi-protein cellular COP9 signalosome complex^26^ and, in view of our two-hybrid data and in vitro binding observations, we wished to ascertain if the interaction between the N-terminal region of Sos1 (NDP peptide) and the COP9 signalosome occurs also in vivo, and the status of cell stimulation under which such an interaction may occur.

For this purpose, we initially used HeLa cells that were transiently transfected with an epitope-tagged (HA) NDP construct. Direct cytoplasmic lysates, or immunoprecipitates thereof using polyclonal rabbit anti-CSN7a antibody or control unspecific IgG, were subsequently probed by immunoblotting with anti-HA antibodies (Fig. 2b). Notice that anti-CSN7a antibody was used here as this antibody is able to immunoprecipate the whole COP9 complex, including its CSN3 component protein^27^ and therefore this approach would not compromise any potential, putative CSN3-Sos1 interaction. These WB data clearly demonstrated the interaction of the COP9 signalosome with the endogenously overexpressed HA-NDP peptide in both serum-starved or serum-stimulated HeLa cells (Fig. 2b).

We also wanted to evaluate the ability of the COP9 signalosome to recognize and interact with endogenous, full-length cellular Sos1 protein (Fig. 2c). In this case, we used Sos2-KO mouse embryonic fibroblasts (MEFs)^28^ so as to exclude any potential interference by the highly homologous Sos2 protein. Cultures of these MEFs were starved before FCS stimulation and then direct cellular lysates or immunoprecipitates of the same lysates using anti-CSN7 antibodies were probed by WB immunoblotting with anti-Sos1 antibodies (Fig. 2c). These assays consistently detected the presence of Sos1 in the COP9 immunoprecipitates. In addition, the amount of co-immunoprecipitated Sos1 appeared to be significantly higher in lysates of serum-stimulated as compared with serum-starved MEFs (Fig. 2c).

Further confirmatory evidence supporting the in vivo interaction between Sos1 and the COP9 signalosome was obtained in similar, complementary assays using antibodies against CSN3 and Sos1 on lysates of cultured mouse NIH3T3 (not shown) or human HeLa cells (Fig. 2d), as well as of primary MEFs derived from WT or conditional, TAM-inducible, Sos1-KO mice (Fig. 2e).

The interaction of Sos1 with the COP9 signalosome suggested the possible processing of cellular Sos1 through the Ubiquitin–Proteasome system (UPS). As ubiquitination is central to the UPS in order to generate degradation signals on substrates destined for destruction^29^, we used immunoblot assays to look for this possible postranslational modification of Sos1 in primary, WT or Sos1-depleted MEFs^30–32^. Our WB data showed that a sizeable portion of the endogenous Sos1 was found ubiquitinated in the lysates of primary MEFs growing under normal conditions in culture (Fig. 2f).

### CSN3 knockdown expression reduces Sos1 protein stability

The COP9 signalosome (CSN) is a conserved protein complex with isopeptidase activity that controls eukaryotic protein degradation through the UPS by regulating the activity of cullin-RING ligase (CRL) families of ubiquitin E3 complexes. Multiple reports document the involvement of CSN in different cellular and developmental processes, including DNA-damage responses or control of gene expression and the cell cycle^33–35^.

**Fig. 3:**
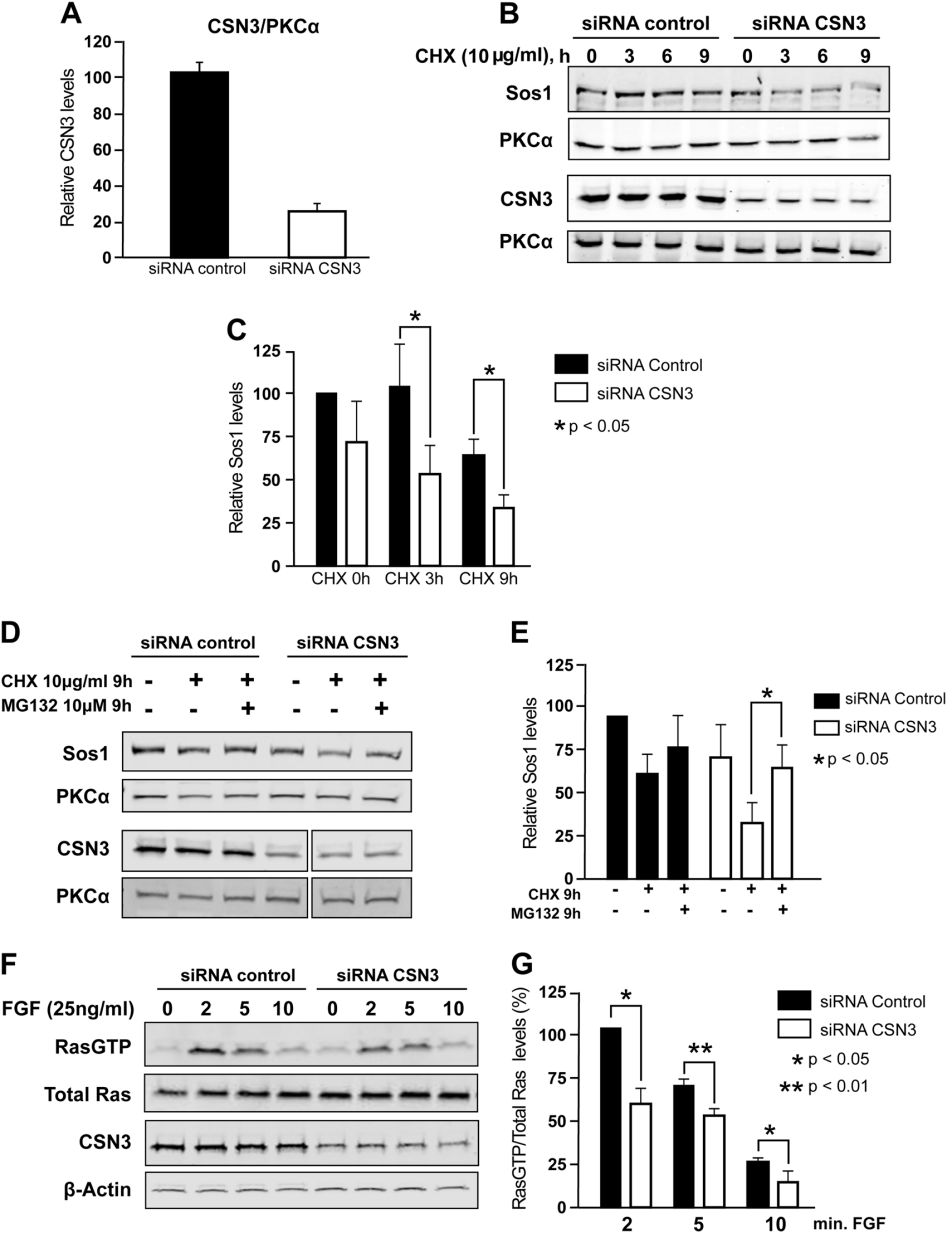
CSN3 depletion decreases the level of mSos1 in MEFs Sos2-KO and inhibition of proteasome prevents this decay. Sos2-KO MEFs were transfected with 60 nM either control siRNA scramble (siRNA control), or a pool of four specific CSN3 siRNA (siRNA CSN3). 48 h after the transfection the cells were treated with cycloheximide 10 µg/ml up to 9 h. **a** Quantitation of CSN3 protein normalized to the PKCα levels, average of three sets of experiments (four time-points each). **b** Endogenous Sos1, CSN3, and PKCα levels were assessed to different times by immunoblotting with specific antibodies after whole-cell extracts were resolved by SDS-PAGE. The result shown is representative of three separate assays. **c** Quantitation of mSos1 protein levels normalized to the PKCα levels. The histograms represent the average and SD of three separate assays. **d** Sos2-KO MEFs were transfected with either 60 nM control siRNA scramble (siRNA control), or specific CSN3 siRNAs (siRNA CSN3). Forty-eight hours after the transfection, the cells were treated as indicated with cycloheximide (10 µg/ml) and/or MG132 (10 µM) up to 9 h. The endogenous levels of Sos1, CSN3, and PKCα were detected by immunoblotting with specific antibodies after the whole cell extracts were resolved by SDS-PAGE. The result shown is representative of three separate assays. **e** Quantitation of mSos1 protein levels normalized to the PKCα levels. The histogram represents the average and SD of three separate assays. **f** RasGTP formation after CSN3 silencing. The level of RasGTP formed after stimulation of CSN3-silenced or control Sos2-KO MEFs treated with FGF (25 ng/ml) for the times (min) indicated were followed by means of immunoblotting using specific anti-RasGTP, anti-pan-Ras, anti-CSN, or anti-B-actin antibodies. **g** Quantitation of RasGTP levels after normalization to total cellular Ras. The histogram represents the average of three separate experiments

To examine whether Sos1 protein stability/homeostasis is affected by the COP9 signalosome, we knocked-down CSN3 expression in MEFs from mSos2-KO mice using specific siRNAs (Fig. 3). Transfection of these MEFs with a pool of four specific siRNAs covering a portion of the mouse CSN3 coding sequence resulted in a significant decrease (about 80%) of endogenous CSN3 expression as compared with MEFs transfected with control, scrambled siRNA (Fig. 3a). To investigate the long-term stability of cellular Sos1, the same mSos2-KO MEFs cells were incubated in absence or presence or cycloheximide (CHX) for up to 9 h, and the decay of the Sos1 protein was followed over time by WB immunoblotting using anti-Sos1 antibody (Fig. 3b). Quantitation of the mSos1 signal in the immunoblots showed that CSN3 silencing was associated with detection of significantly decreased levels of cellular mSos1 protein in the CHX-treated cells, whereas in control cells the mSos1 levels remained basically unaltered (Fig. 3c). These data indicate that Sos1 protein durability diminishes following CSN3 knockdown and suggest that the COP9 signalosome participates in regulation of the stability of cellular Sos1 protein.

As a known function of the COP9 signalosome is the control of proteolysis via the UPS pathway^34,36^, we also wished to test whether the CSN3-dependent stabilization of Sos1 could be mechanistically linked to processing of Sos1 protein through the 26S proteasome pathway. For this purpose, mSos2-KO MEFs cells were treated with proteasome inhibitor MG132 in conjunction with CHX for 9 h, and the rate of Sos1 protein decay was monitored by means of immunoblot analysis (Fig. 3d). Quantitation of the WB data showed that treatment of these MEFs with MG132 for 9 h protected Sos1 from degradation in the presence of CHX. Indeed, when CSN3 expression was silenced by specific siRNAs, the relative Sos1 protein levels were reduced to about 34% as compared with the same MEFs treated with CHX in the absence of MG132 (Fig. 3d, e).

It was also apparent that the reduced cellular levels of Sos1 detected after CSN3 silencing had relevant functional consequences at the level of cellular physiology, as shown by the significant reduction of the rate of RasGTP formation measured upon FGF stimulation in CSN3-siRNA-silenced cells as compared with control mSos2-KO MEFs (Fig. 3f, g).

These observations suggest that the interaction between Sos1 and CSN3 protects Sos1 from proteolytic degradation occurring in cultured MEF cells through the 26 S proteasome pathway, and that this interaction may be functionally relevant.

### CSN3-associated kinase PKD phosphorylates Sos1 protein in vitro

**Fig. 4:**
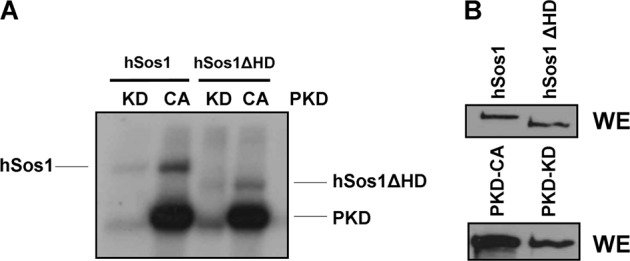
PKD phosphorylates cellular Sos1. **a** HEK293T cells were transfected with 5 µg of AU5-hSos1, AU5-hSos1-ΔHD, pEFBOS-GFP-PKD1-KD, or pEFBOS-GFP-PKD1-CA. After 48 h transfection, all cells were serum-starved for 18 h, and 200 µg of whole-cell extracts were immunoprecipitated with anti-AU5 and anti-PKD antibodies. **b** The expression levels of transfected proteins were detected by immunoblotting with specific antibodies after whole-cell extracts (WE) were resolved by SDS-PAGE. In vitro kinase assays were performed on the immunoprecipates as described in Materials and methods

Like other cellular kinases, protein kinase D (PKD) was reported to be associated to the COP9 signalosome complex^27,37,38^ through its interaction with CSN3^27^. In order to determine whether PKD is able to phosphorylate Sos1, anti-AU5 immunoprecipitates of lysates of HEK293T cells previously transfected transiently with constructs coding for full-length (AU5-hSos1) or HD-truncated (AU5-hSos1-ΔHD) forms of human Sos1 proteins were used as substrates for in vitro kinase assays with PKD-KD (kinase dead) and PKD-CA (constitutively active) mutants of PKD (Fig. 4a). Our assays showed clear phosphorylation of both hSos1 protein forms (both the WT and the mutant lacking the HD domain) in the presence of the constitutively active mutant of PKD (PKD-CA). In contrast, our data showed absence of phosphorylation of the hSos1 protein forms when the assays were performed in the presence of the kinase-dead mutant PKD-KD (Fig. 4a). Consistent with previous reports^39,40^, our assays also detected autophosphorylation of the PKD-CA protein form (Fig. 4a).

Potential matches for the consensus substrate target motif (L/I-X-R-X-X-S/T) recognized by PKD^41,42^ are located within various regions of Sos1, including the REM and CDC25H motifs (perfect matches at positions T605, T789, and S921) or the DH region (imperfect matches at positions S385 and S401). Given the known conformational interactions between the HD and the DH-PH motifs, it will be important to test in future the phosphorylation status of individual mutations at each of those positions to determine the exact mechanistic contribution of their potential phosphorylation to cellular Sos1 stability.

### Knockdown of all cellular PKD forms (1, 2, and 3) reduces Sos1 protein stability

As PKD1 is able to phosphorylate the Sos1 protein in vitro and is also known to associate with the COP9 signalosome via its interaction with CSN3^27^, we wished to explore further the potential in vivo relationship between PDK activity and cellular Sos1 protein homeostasis.

**Fig. 5:**
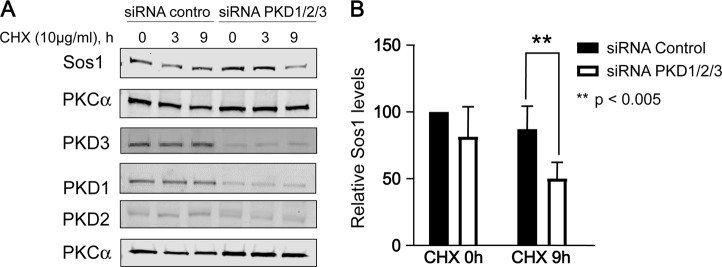
PKD depletion decreases the level of mSos1 in Sos2-KO MEFs. **a** MEFs Sos2-KO cells were transfected with 60 nM control siRNA scramble (siRNA control), or 24 nM siRNAs PKD1 and 24 nM siRNAs PKD3 and 12 nM siRNAs PKD2. After 48 h transfection, the cells were treated with cycloheximide (10 µg/ml) for 9 h. The endogenous mSos1, PKD1, PKD3, and PKCα levels were detected by immunoblotting with specific antibodies after whole-cell extracts were resolved by SDS-PAGE. The data shown are representative of three separate assays. **b** Quantitation of mSos1 protein levels normalized to the PKCα levels. The histogram represents the average and SD of three separate assays

For this purpose, we knocked-down PKD expression in mSos2-KO MEFs using specific siRNAs and then tested the level of Sos1 protein and various related cellular kinases by means of WB immunoassays using specific antibodies (Fig. 5). Our previous qRT-PCR analyses of the expression levels of the three members of the PKD family (PKD1, PKD2, and PKD3) showed high levels of expression of the PKD1 and PKD3 forms, whereas PKD2 was not expressed in these MEFs (data not shown). However, for these experiments, we chose to use siRNAs directed at silencing all three PKD isoforms so as to try and prevent any potential PKD2 expression that might occur as possible rebound/feedback consequence of reducing levels of the other two isoforms. Thus, PKD1/2/3 siRNAs were transiently transfected into MEFs and, after treatment of the transfected cells with CHX for 9 h, the endogenous level of mSos1 protein was evaluated at different time points by WB using anti-Sos1 antibody (Fig. 5a). Quantitation of our data clearly showed that, after 9 h of protein synthesis blockade, the level of mSos1 protein in the PKD-knocked-down MEFs cells was significantly decayed (about 50%) in comparison with cells transfected with control siRNA scramble (Fig. 5b), indicating that COP9 signalosome-associated-PKD kinase plays a role in in vivo regulation of mSos1 protein stability in these cells.

## Discussion

The proteins of the mammalian Sos family are composed by a linear sequence of functionally distinct modular domains. In this report, we focused on the N-terminal region of Sos1, encompassing the histone-like domain (HD), the Dbl-homology domain (DH), the pleckstrin domain (PH), and the helical linker (HL)^5,6^. A role of this region in intramolecular regulation of Sos GEF activity has been previously recognized^18,19^, but its overall functional relevance is not yet fully understood, and various regulatory questions concerning the domains within this region remain unclear.

Looking for cellular proteins with putative binding affinity for the N-terminal region of Sos1, we carried out a yeast two-hybrid screening of a HeLa cDNA expression library and found strong positive interaction between CSN3 and the Sos1 histone-like domain (HD). This binding was confirmed in vitro by pull-down experiments with GST-fusion proteins and in vivo by means of co-immunoprecipitation assays in lysates of mouse and human cell lines, thus confirming the occurrence in eukaryotic cells of direct physical interaction between CSN3, a subunit of the COP9 signalosome, and the HD motif of Sos1. Further studies will be needed in future to pinpoint more accurately the exact region within the HD that is involved in this interaction and also to ascertain whether the highly homologous Sos2 proteins^43^ are also able to interact with the COP9 signalosome.

The HD contains two tandem histone folds and exerts negative control on the overall Ras-GEF activity of Sos1 under basal conditions^6^. Previous structural studies showed that the HD is conformationally coupled to the DH-PH unit, therefore stabilizing the auto-inhibited conformation of Sos1, in which the DH-PH domain is also known to interfere with the allosteric binding motif for Ras^17,22^. Only upon ligand stimulation of upstream tyrosine kinase receptors (RTK), the second phase of Sos activation—(i) recruitment to the plasma membrane (involving PH binding to membrane phosphoinositides and electrostatic interaction between a positively charged patch of the HD and the negatively charged membrane) and (ii) disruption of the auto-inhibited conformation mediated by the DH-PH unit— can take place. Once in the membrane, the reoriented Sos molecules, displaying increased accessibility to both of its allosteric and catalytic Ras binding sites, can processively catalyze nucleotide exchange on multiple Ras molecules. Various mutations associated with Noonan syndrome, a disease caused by hyperactivated Ras signaling, are located in the HD and the PH domains^6,17,22,44,45^. Intramolecular interaction between the HD and the HL, located between PH and REM domains, may also contribute to maintain the inactive, auto-inhibited conformation of Sos since a mutation in the HL (R552G) increases the nucleotide dissociation rate of Ras and the mutated HL does not interact with the HD^22,46,47^. It will be interesting to determine in future whether or not any of the known Noonan HD mutations may affect the Sos1-CSN3 interaction in vivo.

Our observations characterizing the interaction between CSN3 and the Sos1 HD suggest that this domain not only functions regulating Sos-GEF autoinhibition but is also involved in other functional roles, such as the control of Sos protein stability and homeostasis by modulating the degradation and intracellular levels of Sos1. On one side, our data showed that an in vivo interaction occurs between the COP9 signalosome and endogenous full-length Sos1 protein and that such an interaction is probably functionally significant as the interaction between them is higher upon RTK activation, probably due to disruption of the auto-inhibited conformation resulting from competition with, or uncoupling of, the basal HD/DH-PH interaction. On the other hand, our two-hybrid and co-immunoprecipitation experiments demonstrating physical interaction between the CSN3 and Sos1 support the notion of the participation of the UPS pathway in regulation of the homeostasis and intracellular levels of Sos1 in eukaryotic cells.

The COP9 signalosome(CSN) is a highly conserved, nine-subunit (CSN1–9) protein complex involved in the ubiquitin–proteasome system in eukaryotic organisms^25,34^. CSN serves as a dominant regulator of cullin-RING ligase family of ubiquitin E3 complexes (CRLs)^48^ through removal of Nedd8 from cullins^26,48,49^. All CSN subunits are essential for full function of the complex and are produced in free-form before coordinated complex assembling^25,26^. Heterozygous deletion of CSN3 is the molecular cause of Smith–Magenis syndrome that results in reduction of the amount of the CSN protein complex^50^, according to the functional role of CSN3 for the CSN complex stabilization^33^.

The CSN is associated with several protein kinases (CK2, AKT, ATM, PKD) able to modify substrates of the UPS, therefore affecting the stability of different cellular proteins^25,27,51,52^. Specifically, PKD interacts with full-length CSN3 and modifies CSN7^27^. In addition, CSN-associated PKD phosphorylates c-Jun and p53; CSN-mediated phosphorylation targets p53 for degradation by the UPS but stabilizes c-Jun, and inhibition of CSN-associated kinases triggers ubiquitin-conjugate formation and c- the case of Sos1, our data indicate that CSN3-associated PKD is able to phosphorylate Jun degradation^27^. Therefore, CSN acts as a platform recruiting protein kinases for regulating ubiquitin-conjugate formation by different E3 ubiquitin ligases^25,27^. In the case of Sos1, our data indicate that CSN3-associated PKD is equally able to phosphorylate the full-length Sos1 or a mutant lacking the HD domain. We also found that knockdown of all PKD forms or of CSN3, which destroys the CSN complex^33^, increases Sos1 degradation by the 26S-proteasome pathway. Future mechanistic studies will be needed to ascertain whether PKD mediates Sos1 stabilization through direct phosphorylation of the Sos1 protein at any of its conserved target sites for this kinase or through its interaction with CSN3 or other components of the COP9 signalosome. As several consensus PKD phosphorylation sites for Sos1 (positions T605, T789, and S921) are also conserved in Sos2, these studies will also be relevant to analyze the possibility of dual control of the stability of both Sos1/2 isoforms via signalosome/PKD action.

Taken together, our results strongly suggest that the CSN/PKD complex is involved in stabilization of Sos1 proteins. Although regulation of CSN-dependent deneddylation activity^25,27^ could be a potential mechanism, the exact mechanistic details responsible for such a stabilization effect remain to be clarified by future studies. In any event, our data are consistent with the oncogenic role proposed for CSN^53^ and the CSN3 protein^54^ in different tumor types, and highlight the possibility that the CSN/PKD complex might be a potential molecular target for design of new therapeutic drugs.

## Materials and methods

### Cell lines, transfections, antibodies, and reagents

NIH3T3 cells were maintained in Dulbecco’s modified Eagle’s medium (DMEM; Invitrogen, Paisley, UK) supplemented with 10% calf serum (CS, Invitrogen). Human HeLa cells, HEK293T cells, and mouse embryonic fibroblasts MEFs (WT, Sos1-KO, Sos2-KO, and Sos1/2-DKO)^28,30^ were maintained in DMEM supplemented with 10% fetal calf serum (FCS; Invitrogen). Before immunoprecipitation, Sos1-KO and DKO cells were treated for 9 days with 0.3 µM tamoxifen before lysis. Cultures were tested routinely to exclude mycoplasma contamination (Universal Mycoplasma Detection kit, ATCC, Manassas, VA, USA; #30-1012). Transient transfections of HeLa and HEK293T cells were performed in p100 plates using Jet-Pei (Polyplus-Transfection, Illkirch, France). For serum-starvation cells received DMEM containing 0.5% FBS 24 h after transfection and were then further incubated for 18 h. Cells were stimulated with 30% FCS for 10 min. All assays were done 48 h after transfection. Monoclonal mouse antibodies against CSN3, ubiquitin (clone P4D1, sc-8017) and Sos1, and polyclonal rabbit antibodies against CSN7a, Sos1 (sc-256), Sos2 (sc-258), PKD1, and GST, were purchased from Santa Cruz Biotechnology (Santa Cruz, CA, USA). Monoclonal rabbit antibody anti-PKCα, clone M4 was purchased from Millipore; anti-HA and anti-AU5 monoclonal antibodies from Berkeley Antibody Company (Berkeley, CA); polyclonal rabbit antibodies against PKD2 and PKD3 from Cell Signaling; and polyclonal rabbit antibody against CSN3 was acquired from Abcam (Ab79698-Cambridge, UK). Bound antibodies were detected in some experiments with secondary antibodies conjugated with IRDye680 or IRDye800 and analyzed with an Odyssey Imager system (LI-COR, Lincoln, NE), or anti-mouse or anti-rabbit horseradish peroxidase (1:5000; Bio-Rad) used as a secondary antibody were visualized using an Enhanced Chemiluminescence Detection Kit (Amersham, Arlington Heights, IL, USA). 5-Bromo-4-chloro-3-indolyl β-D-galactopyranoside (X-Gal), 4-hydroxy-tamoxifen(TAM) (H7904), Cycloheximide (C1988) and MG132 (M7449) were from Sigma Co. (St. Louis, MO, USA). [γ32P]-ATP (370 MBq/ml) was from PerkinElmer, Inc. (Boston, MA, USA).

### DNA constructs

The majority of hSos1 constructs were described previously^55^. pCEFL-KZ-AU5-hSos1 was cloned by amplifying hSos1 with specific primers providing Bcl-I and Sma-I sites at the 5ʹ and 3ʹ ends, respectively, and the amplified product was subcloned into Bgl-II and Eco-RV sites in pCEFL-KZ-AU5. To obtain HD-truncated mutant of hSos1 (pCEFL-KZ-AU5-hSos1-ΔHD), plasmid pCEFL-KZ-AU5-DH-PH was digested with Hind-III and Spe-I, and the AU5-DH-PH fragment was subcloned into Hind-III and Spe-I sites of pCEFL-KZ-AU5-hSos1. To previously generate pCEFL-KZ-AU5-DH-PH, the DH-PH domain of hSos1 (coding region 537–1653) was PCR-amplified from the pCEFL-KZ-HA-hSos1 plasmid using specific primers and providing sites BglII and NotI at the 5´ and 3´ ends, respectively. The amplified product was then subcloned into BglII and NotI sites of vector pCEFL-KZ-AU5^55^. The HD and NDP domains of hSos1 were PCR-amplified using the specific primers (corresponding to coding regions 1–640 and 1–1790, respectively) providing sites Eco-RI and Sal-I at the 5ʹ and 3ʹ ends, respectively. The amplified products were then subcloned into Eco-RI and Xho-I sites in pGEX-4T-1 (Amersham Biosciences). The DH-PH domain of hSos1 (coding region 537–1653) was PCR-amplified using specific primers and providing sites Eco-RI and Not-I at the 5ʹ and 3ʹ ends. The amplified products were then subcloned into Eco-RI and Not-I sites in pGEX-4T-1. pEFBOS-GFP fused to PKD1 kinase-dead (single mutant Asp733Ala; PKD1KD) or constitutively active (double mutant Ser744/748Glu; PKD1CA) were a gift from T. Iglesias^56^. The sequences of the oligonucleotides utilized are available upon request.

### Yeast two-hybrid system

PCR-derived fragments encoding for the appropriate NDP, HD, DH-PH, DH, PH, and PRII domains (hSos1 coding regions 1–1790, 1–640, 537–1653, 537–1326, 1263–1701, and 1–1790, respectively) were subcloned into plasmid pEG202, encoding the LexA-binding domain. The full-coding sequence for CSN3 was cloned into plasmid pJG45, encoding an amino-terminal B42-activation domain. *Saccharomyces cerevisiae* EGY48 strain (MATα, his3, trp1, ura3-52, leu2: pLEU2-LexAop6) was used as a host. Co-transformation of two-hybrid vectors into yeast were performed as described^57^. Protein–protein interaction in the two-hybrid system was monitored as described^55^.

### Immunoprecipitation

Cells treated as indicated in each case were lysed in lysis buffer and nucleus-free supernatants were incubated with appropriate antibodies as described^55^. Immune complexes were collected by centrifugation, washed five times with lysis buffer, resolved by SDS-PAGE and visualized by immunoblotting.

### Bacterial expression of fusion proteins

GST-fusion proteins were obtained and purified as described^55^. In all binding experiments, transfected mammalian cells were lysed in lysis buffer^55^ and 20 μg of the corresponding GST-fusion proteins, coupled to glutathione–sepharose beads, were used^55^.

### RNA interference

In knockdown experiments, siRNA duplexes (60 nM) were transfected twice in MEFs using Lipofectamine RNAiMAX Reagent (Invitrogen) at 24 h intervals. To knockdown CSN3 and PKD1/3, we used siGENOME SMART pool mouse Cops3 (M-047361-00), siGENOME SMART pool mouse Prkd1 (M-048415-01), siGENOME SMART pool mouse Prkd2 (M-040693), siGENOME SMART pool mouse Prkd3 (M-040692-00), and for siRNA control siGENOME control pool (D-001206-14-20), all purchased from Dharmacon.

### In vitro kinase assays

In vitro kinase assays were performed as described^58^ by incubating immunoprecipitated kinase (GFP-PKD-KD or GFP-PKD-CA) with eluted substrates or AU5-hSos1 or AU5-hSos1-ΔHD immunoprecipitates in the presence of ATP-gamma P^32^.

### Statistical analysis

Data were analyzed using GraphPad Prism 7.0. Results expressed as mean ± SD of the indicated number of experiments. Statistical significance was evaluated using the *t* test for unpaired observations. Western blots were analyzed using linear correlations between increasing amounts of protein and signal intensity.
